# A novel nasal co-loaded loratadine and sulpiride nanoemulsion with improved downregulation of TNF-α, TGF-β and IL-1 in rabbit models of ovalbumin-induced allergic rhinitis

**DOI:** 10.1080/10717544.2021.1872741

**Published:** 2021-01-27

**Authors:** Soad A. Mohamad, Mohamed A. Safwat, Mahmoud Elrehany, Sherif A. Maher, Ahmed M. Badawi, Heba F. Mansour

**Affiliations:** a Department of Pharmaceutics, Faculty of Pharmacy, Deraya University, Minia, Egypt; b Department of Pharmaceutics, Faculty of Pharmacy, South Valley University, Qena, Egypt; c Department of Biochemistry, Faculty of Pharmacy, Deraya University, Minia, Egypt; d Department of Biochemistry, Faculty of Medicine, Minia University, Minia, Egypt; e Department of Otorhinolaryngology, Faculty of Medicine, Minia University, Minia, Egypt; f Department of Pharmaceutics, Faculty of Pharmacy, Minia University, Minia, Egypt

**Keywords:** Loratadine, sulpiride, nasal, nanoemulsion, rhinitis

## Abstract

**Purpose:**

The work aimed to develop a co-loaded loratadine and sulpiride nasal nanoemulsion for allergic rhinitis management.

**Methods:**

Compatibility studies were conducted adopting differential scanning calorimetry and Fourier transform infrared spectroscopy. Nanoemulsion formulations were prepared using soybean lecithin, olive oil and tween 80. Sodium cholate and glycerol were employed as co-surfactants. Nanoemulsions were assessed for viscosity, pH, droplet size, polydispersity index, zeta potential, electrical conductivity, entrapment, *In vitro* drug release and corresponding kinetics. Stability of the selected formulation was investigated. The biological effectiveness was evaluated in rabbit models of ovalbumin-induced allergic rhinitis by measuring TNF-α, TGF-β and IL-1.

**Results:**

Compatibility studies revealed absence of drug/drug interactions. Nanoemulsions exhibited > 90% entrapment efficiency. The selected nanoemulsion demonstrated small droplet size (85.2** **±** **0.2** **nm), low PDI (0.35** **±** **0.0) and appropriate Zeta Potential (−23.3** **±** **0.2) and stability. It also displayed enhanced in vitro drug release following the Higuashi Diffusion and Baker–Lonsdale models. The mean relative mRNA expression of TNF-α, IL-1 and TGF-β significantly decreased from 9.59** **±** **1.06, 4.15** **±** **0.02 and 4.15** **±** **0.02 to 1.28** **±** **0.02, 1.93** **±** **0.06 and 1.56** **±** **0.02 respectively after treatment with the selected nanoemulsion formulation.

**Conclusion:**

The results reflected a promising potent effect of the combined loratadine and sulpiride nasal nanoemulsion in managing the symptoms of allergic rhinitis.

## 1. Introduction

Allergic rhinitis is a disease provoked by IgE-mediated immune response and demonstrates a long-lasting inflammation of nasal mucosa. IgE/allergen interaction on the exterior of basophils and mast cells results in the stimulation of these cells for the liberation of mediators comprising histamine, leukotrienes, prostaglandins, platelet activating factor (PAF) and cytokines. T-helper cell (Th2) cytokines are known to have a principal responsibility in the developing allergic sensitization and pathology of allergic inflammation (Maes et al., 2012). Furthermore. IL-1, TNF-α, and TGF-β cytokines are commonly exist in the inflamed sites in the body. These mediators can consequently recruit extra inflammatory cells, initiate the release of more inflammatory mediators and stimulate afferent nerves (Greiner et al., 2011). Moreover, it has been reported that this condition is accompanied by increase of lysophosphatidyl choline that in turn increases the cell permeability to sodium (Na^+^) and calcium (Ca^2+^), induces membrane depolarization, enhances IgE response, supports phagocytic action, prevents adenylate cyclase, and stimulates phosphodiesterase and consequently reducing cAMP levels in the cells. These changes promote the liberation of mediators from mast cells generating airway inflammation (Agrawal et al., 1986). The released mediators in this allergic condition trigger allergic manifestations (Wang et al., 2016) including nasal symptoms such as rhinorrhoea, sneezing, nasal itching and nasal obstruction (Aria Workshop Group; World Health Organization, 2001). Eye redness, itching and tearing can also develop. As the related symptoms may disrupt sleep, cause lethargy, and affect patient concentration, it has been reported that this type of allergy affects the quality of patient life and adds loads on the health care systems (Kim et al., 2007). The usual pharmacological therapeutic protocol for the management of allergic rhinitis involves the use of topical and/or oral antihistamines, intranasal corticosteroids, anticholinergics or antihistamine-decongestant combinations, leukotriene receptor antagonists and mast cell stabilizers such as cromoglycic acid (Sur & Scandale, 2010). As histamine has the major role in the allergic reactions, this type of rhinitis is commonly treated with antihistamines that have been clinically used for several years. Antihistamines manage the baseline symptoms including sneezing and nasal secretions and inhibit the allergen-provoked liberation of mediators from mast cell in the nasal mucosa. As histamine release is responsible for all the pathological features of allergic rhinitis except the inflammatory reactions of the late phase (Iriyoshi et al., 1996), patients do not attain entire symptom management with a single drug therapy (Fabbri et al., 2014). Thus, combination treatment can be recommended for managing the entire symptoms of the condition.

The second-generation antihistamine, loratadine is known to be used in the treatment of allergic rhinitis. The drug has good absorption from the gastrointestinal tract when given orally reaching peak plasma concentration after 1 to 1.5** **hour (Moffat et al., 2004). However, it has poor oral bioavailability (40%) as it experiences rapid first-pass hepatic metabolism (Borgaonkar et al., 2011). Therefore, in order to avoid the drug metabolism in the liver, an alternate route of delivery would be favored.

Sulpiride, an antipsychotic drug, is a selective dopamine receptor antagonist. It has high affinity to D2 and D4 receptors. It has been reported that sulpiride has an anti-inflammatory effect through increasing the intracellular cAMP level regardless the presence of dopamine signaling at D2 receptors (Brustolim et al., 2006). Based on this finding, it can be speculated that the drug can have a beneficial effect in the management of allergic rhinitis inflammatory manifestations. Sulpiride experiences a limited oral bioavailability not more than 27% due to its poor water solubility (Zidan et al., 2015).

The nasal route represents a promising noninvasive alternative route of drug delivery for treatment of different conditions. Rapid absorption of drugs into systemic circulation is permitted through the porous endothelial membrane of the lush vascular capillary layer beneath the nasal mucosa (Illum, 2002). Moreover, nasal delivery improves poor bioavailability and avoid hepatic first pass metabolism and drug degradation in gastrointestinal tract (Khan et al., 2018). This route is adopted also for local drug delivery to avoid the systemic exposure to certain drugs and reduce the related side effects (Djupesland et al., 2013).

Nanoemulsions are colloidal systems consisting of two immiscible phases. They are translucent having droplet size around 200** **nm. These systems are kinetically stabilized by the aid of surfactants or a mixture of surfactants and co-surfactants (Gurpreet and Singh, 2018). Nanoemulsions have the advantages of controlling the drug release and the possibility of delivering a wide variety of therapeutic agents (Hoeller et al., 2009). Additionally, they are superior over macroemulsion in having larger surface area and free energy. They also avoid coalescence, flocculation, creaming and sedimentation. Nanoemulsions can be prepared adopting lower concentration of emulsifying agents and thus reducing surfactant-related toxicity (Liu et al., 2011). Furthermore, these systems have the ability to solubilize poorly water-soluble drugs and thus improve their permeation through mucosa and provide an advantageous formulation for delivering such drugs (Gaba et al., 2019).

The present work, represent for the first time the co-loading of loratadine and sulpiride into nanoemulsion for nasal delivery as a new approach with enhanced therapeutic effect for the management of allergic rhinitis. Lecithin based nanoemulsions of combined loratadine and sulpiride were formulated. The developed nanoemulsions were investigated for in vitro characterization. Biological studies have been conducted to evaluate the efficacy of the selected formulation in ovalbumin-induced allergic rhinitis rabbit models.

## 2. Materials and methods

### 2.1. Materials

Loratadine (BP), sulpiride (BP), Olive oil (highly refined), Sodium deoxycholate, Soya bean phsphatidylecholine (EP), Ovalbumin (Grade V) and cellulose membrane (molecular weight cutoff 12000– 14000) were bought from Sigma–Aldrich Chemical Co. (Poole, UK). Tween 80 (chemically pure), Al (OH)_3_ and Glycerol were obtained from Fisher Chemical (Loughborough, UK). Other chemicals and reagents were of analytical grade and purchased from El-Nasr Pharmaceutical Company (Cairo, Egypt).

### 2.2. Investigation of loratadine/sulpiride compatibility

#### 2.2.1. Fourier transform infrared spectroscopy

Fourier transform infrared (FTIR) spectra of loratadine, sulpiride and their physical mixture (1:1) were inspected using a FTIR spectrometer (Perkin Elmer, NY, USA). 5** **mg samples was squashed into disks of potassium bromide. The spectra were attained at the wavelength range from 500 to 4000** **cm^−1^.

#### 2.2.2. Differential scanning calorimetry study

Differential scanning calorimetry (DSC) examination of loratadine, sulpiride and their physical mixture (1:1) were performed using a DSC-131 Evo (Setaram Inc., France). 2–5** **mg samples were weighed in crucible pans covered with pierced caps. The temperature was raised gradually from 25 to 500** **°C at a heating rate of 10** **°C/min under a nitrogen flow rate of 40** **ml/min.

### 2.3. Preparation of the co-loaded loratadine and sulpiride nanoemulsions

Combined loratadine and sulpiride nanoemulsions (o/w) was developed adopting ultra-sonication technique. Different compositions of the developed formulations are shown in Table 1. Specified amounts of Tween-80 and soybean lecithin were mixed at 25 °C using a magnetic stirrer (400** **rpm for 30** **minutes) to form the oil phase. The specified amounts of olive oil (3.5** **g), loratadine (0.25** **g) and sulpiride (0.05** **g) were added and stirred till obtaining a uniform mixture. The calculated amounts of deionized water, sodium cholate and glycerol (co-surfactants)were mixed to form the aqueous phase. The oil phase was then added gradually to the aqueous phase using a suitable syringe under stirring for 10** **minutes. The resultant mixture was further sonicated for 30** **minutes adopting ultrasonic processor at 20** **kHz (FB-110Q, Shanghai Litu Machinery and Equipment Engineering Co., LRD, Shanghai, China).

**Table 1.** Compositions of the co-loaded loratadine/sulpiride nanoemulsion formulations.

Formulation	Loratadine % w/v	Sulpiride % w/v	Olive oil % v/v	Tween 80 % v/v	Sod cholate % w/v	Phospholipid % w/v	Glycerol % v/v	water q.s. (ml)
F1	0.5	0.1	7	2	_	5	2.5	50
F2	0.5	0.1	7	–	2	5	2.5	50
F3	0.5	0.1	7	2	1	5	2.5	50
F4	0.5	0.1	7	2	2	5	2.5	50
F5	0.5	0.1	7	2	1	5	_	50
F6	0.5	0.1	7	2	1	7	2.5	50
F7	0.5	0.1	7	2	1	2.5	5	50

### 2.4. Characterization of the prepared nanoemulsions

#### 2.4.1. Appearance, viscosity and pH

The prepared nanoemulsions were visually examined by inspecting the transparency on light reflections. Their pH was determined using a pH meter (model 361, Systronics). Viscosity of the prepared nanoemulsions were investigated using a Brook Field Viscometer (LVF 69726) with a UL-adapter. All the investigations were accomplished in triplicate at 25 °C.

#### 2.4.2. Entrapment efficiency

Centrifugation method was employed to establish the entrapment efficiency of the co-loaded loratidine and sulpiride nanoemulsion (Prabhakar et al., 2013). Briefly, 2.0** **mL sample was centrifuged at 3500** **rpm for 15** **minutes. The aqueous phase or the supernatant was separated, while the residue was washed two times with distilled water and recentrifuged to ensure entire separation of the free drugs. The separated supernatants were added together, mixed with equivalent volume of ethanol, and mixed for five minutes using a vortex mixer. The amount of free loratadine and sulpiride was established by measuring the spectrophotometric absorbance at 395 and 218** **nm respectively using a-1900PC UV-Vis spectrophotometer (Shanghai Puyuan Instrument Co., LRD, China) and adopting ultraviolet spectroscopy chemometric technique (Gad et al., 2013; Sood et al., 2014). The drug amounts entrapped in the oil phase were estimated by calculating the difference between the total drug amount incorporated and the free drug amount detected in the aqueous phase. Entrapment efficiency (%EE) was computed using the subsequent equation:

$$\mathrm{EE}\;(\%)\;=\;\frac{W\;\mathrm{entrapped}\;\mathrm{drug}}{W\;\mathrm{initial}\;\mathrm{drug}}\times \;100$$

#### 2.4.3. Droplet size, polydispersity index, zeta potential and electrical conductivity

The droplet size, polydispersity index (PDI) and zeta potential of the selected nanoemulsions were investigated through dynamic light scattering adopting N4Plus submicron particle size analyzer (Beckman Coulter, UK). Samples were appropriately diluted with distilled water and measured at room temperature. The average values of three measurements ± SD were recorded. A conductivity meter (CM 180) was used to determine the electrical conductivity of the selected nanoemulsions by direct dipping of electrode into the sample at 25 °C.

#### 2.4.4. *In vitro* release studies

To study the *in vitro* release of loratadine and sulpiride from the prepared nanoemulsions, a dialysis bag method was adopted (Sood et al., 2014). 4** **mL sample was loaded into a dialysis bag of cellulose membrane (14** **kDa molecular weight cutoff). The bag was dipped in a beaker containing 100** **ml phosphate buffer solution, pH 6.8** **±** **0.5 kept at 37** **±** **0.1** **°C and stirred at 50** **rpm for 12** **hours. 2** **ml samples were removed at appropriate time intervals. Loratadine and sulpiride amounts were determined by measuring spectrophotometric absorbance at 395 and 218** **nm respectively. The resultant release profiles were compared to those obtained from the individual raw loratadine and sulpiride. Statistical analysis of the release data has been carried out using similarity factor, f2 whereas the release profiles were compared to a reference (Shah et al., 1998).

To define the release mechanism of loratadine and sulpiride from the selected nanoemulsions (F2, F3), data was fitted into different kinetic models:

Zero order *R*=*K*0*t* (Sood & Panchagnula, 1998)
$\text{First order:}\;R=1-e^{-k1}t$ (Carbinatto et al., 2014)
$\text{Higuchi diffusion model:}\;Q=KH\times t^{1/2}$ (Higuchi, 1963)
Baker–Lonsdale model:  3⁄2[1−(1−Mt⁄M∞) 2⁄3]−Mt⁄M∞)= K3t (Baker & Lonsdale, 1974)
$\mathrm{Hixson}–\text{Crowell cube root law:}\;{UR}^{1/3}=k_{4}t$ (Chawla et al., 2000)

Whereas R, Q or Mt/M∞ refers to the fraction of drug released at time t, K or KH is the rate constant related to each model, UR is the unreleased fraction of the drug while n is the diffusional exponent that characterizes the type of release mechanism during the dissolution process.

#### 2.4.5. Morphology analysis

The morphology of the selected nanoemulsion (F3) was investigated through transmission electron microscope (JEM-2100, JEOL, Japan). The selected nanoemulsion was suitably diluted with deionized water and dripped on a copper mesh. For enhancing the image quality, 2% w/v phosphotungstic acid solution was added as a negative stained standard (Mallick et al., 2020). The morphology was also further confirmed via scanning electron microscope. The samples were positioned on polycarbonate substrate while the excess water was removed by drying first at ambient temperature and then by carbon dioxide. Samples were coated with gold and examined under a scanning electron microscope (KYKY EM3200, China) running at an accelerating voltage of 20** **kV.

#### 2.4.6. Stability study

The selected nanoemulsion F3 was stored at 4 °C and 25 °C for one month. The stored nanoemulsion was investigated for changes in droplet size, PDI and % EE (Ghosh et al., 2013).

#### 2.4.7. Statistical analysis

All investigations were conducted in triplicate and by freshly prepared samples. Statistical analysis of data was accomplished using graph pad Prism 8.3 computer software (Graph Pad Software San Diego, CA). All investigational data were conveyed as mean ± standard deviation (SD). One-way ANOVA was used for analysis of the results considering the difference was statistically significant when *p* value < .05 to be.

### 2.5. *In vivo* efficacy study in rabbit models of ovalbumin-induced allergic rhinitis

The study was permitted by the Committee of Animal Ethics in Minia University, Minia, Egypt, that guaranteed the of animals corresponded to the National Institutes of Health guide for the use and care of laboratory animals (NIH Publications No. 8023, revised 1978).

#### 2.5.1. Induction of allergic rhinitis

Allergic rhinitis rabbit models were developed according to a modified reported protocol (Sagit et al., 2017). The rabbits were sensitized via intraperitoneal (IP) injections of 1** **ml freshly prepared ovalbumin every other day over 7** **days followed by intranasal administration of 1** **ml physiological saline (PS) having 0.3** **mg ovalbumin and 30** **mg Al(OH)_3_ for 14** **days (Sagit et al., 2017; Senturk et al., 2018). Treatment was initiated after the 21** **days of the induction and for 14** **days. The studied formulations were administered as 70** **μl dose “holding 17** **μg of the loratadine and 7** **μg of sulpiride” in every nostril by means of a micropipette with a low-density polyethylene tubing, having an internal diameter of 0.1** **mm at the administration site.

#### 2.5.2. Study groups and drug treatment

Twenty-five male New Zeeland rabbits weighing 1.5–2** **kg were used. The rabbits were maintained at room temperature and had access to typical laboratory diet and water. The rabbits were allocated into five groups of five rabbits each:

Group I: Negative control (normal) in which rabbits were injected with 1** **ml intraperitoneal PS followed by intranasal administration of 1** **ml PS.
Group II: Positive control group in which rabbits were sensitized with intraperitoneal ovalbumin and provoked with intranasal ovalbumin but did not receive treatment.
Group III: placebo in which rabbits were sensitized and provoked with ovalbumin and treated with drug-free nanoemulsion.
Group IV: In which rabbits were sensitized and provoked with ovalbumin and treated with intranasal co-loaded loratadine and sulpiride conventional emulsion.
Group V: In which rabbits were sensitized and provoked with ovalbumin and treated with intranasal co-loaded loratadine and sulpiride nanoemulsion (F3).

#### 2.5.3. Evaluation of allergic rhinitis symptoms

The early and late response of the rabbit models after rhinitis induction and intranasal treatment was assessed on the first and fourteenth day of treatment by otorhinolaryngology specialist. Allergic rhinitis was assessed concerning the severity of the classic clinical symptoms including nasal irritation, sneezing, nasal secretions and conjunctivitis and eye secretions. The rhinitis sign scores were classified into a four-point scale, ranged from 0 to 3. For motion of nasal itching: 0** **=** **no nose rubbing; 1** **=** **2 nose rubbing/min; 2** **=** **4–6 nose rubbing/min; while 3 = > 6 nose rubbing/min. For sneezing: 0** **=** **none; 1** **=** **1–3/10** **min; 2** **=** **4–9/10** **min; while 3** **=** **more than 10 sneeze/10** **min. For nasal mucus: 0** **=** **no mucus; 1** **=** **mucus inside a nostril; 2** **=** **mucus outside a nostril; while 3** **=** **overflowing. For eye secretions: 0** **=** **none; 1** **=** **inside eye; 2** **=** **outside eye; while 3** **=** **overflowing or change in color. For conjunctivitis: 0** **=** **none; 1** **=** **mild; 2** **=** **moderate; while 3** **=** **severe. On the last day of sensitization or day 1, the predominant signs including nasal rubbing, sneezing, and nasal secretion were scored to start treatment. The allergic rhinitis model succeeded when the total sign score was more than 7 while the treatment succeeded when the total sign score was less than 4 ( Zhou et al., 2009; Senturk et al., 2018).

#### 2.5.4. Measurement of inflammatory parameters

Different inflammatory parameters were determined prior and post treatment to assess the efficacy of the selected nanoemulsion (F3). These parameters include TNF-α that is a powerful pro-inflammatory cytokine having fundamental responsibilities in stimulating leukocyte staffing to injured spots via provocation of expression of inflammatory chemokines and adhesion molecules. Another inflammatory parameter is TGF-β that is known as a very effective immunosuppressive and anti-inflammatory cytokine, conflicting TNF-α activities, stimulating the production of T regulatory cells, and facilitating the anti-inflammatory activities of these cells **(**Ohno et al., 1992**;** Hamaguchi et al., 1994**;** MULLOU et al., 1995). Interleukin-1 (IL-1) also is a cytokine that is essential for triggering the inherent immune response, facilitating the staffing, stimulation, and adherence of phagocytes (macrophages and neutrophils), and ending the inherent immune response is the same as TNF-α (Ott et al., 2007).

#### 2.5.5. Total RNA extraction from sino-nasal mucosa

About 100** **mg of Sino-nasal tissue was homogenized for extraction of total RNA by ultrasonic homogenizer (Sonics-Vibracell, Sonics & Materials Inc., Newtown, USA) in 1** **ml TRIzol reagent (Amresco, Solon, USA). The entire RNA concentration was determined at A260 nm and the purity was calculated according to the ratio A260/A280. Samples have a purity ≥ 1.7 was used for qRT-PCR using GAPDH (Glyceraldehyde-3-phosphate dehydrogenase) as a reference housekeeping gene for determination of the relative expression of IL-1, TGF-β, and TNF-α.

#### 2.5.6. Real-time qRT-PCR

cDNA synthesis was performed for equal quantities of total RNA in all samples employing the kit of RevertAid H Minus First Strand cDNA Synthesis (#K1632, Thermo Scientific Fermentas, St. Leon-Ro, Germany). Real-time PCR was performed with single-stranded cDNAs. PCRs were accomplished by SYBER Green [#K0251, Thermo Scientific Fermentas St. Leon-Ro, Germany-Maxima SYBER Green qPCR Master Mix (2X)] using the detection system of StepOne Real-Time PCR (Applied Biosystems). The set of primers used for IL-1 *(*GenBank accession NC_013670.1) were as follows: forward primer was 5′-AGCTTCTCCAGAGCCACAAC-3′, and reverse primer was 5′-CCTGACTACCCTCACGCACC-3′. Primers for GAPDH (GenBank accession NC_013676.1) were 5′-GTCAAGGCTGAGAACGGGAA-3′(forward primer) and 5′-ACAAGAGAGTTGGCTGGGTG-3′(reverse primer), and primers for TGF-β (GenBank accession NC_013672.1) were 5′-GACTGTGCGTTTTGGGTTCC-3′ (forward primer) and 5′-CCTGGGCTCCTCCTAGAGTT (reverse primer). primers for TNF-α (GenBank accession NC_013680.1) were 5′-GAGAACCCCACGGCTAGATG-3′(forward primer) and 5′-TTCTCCAACTGGAAGACGCC-3′ (reverse primer). Real-time polymerase chain reaction (qRT-PCR) was performed using 20** **μl of RealMOD Green qRT-PCR Mix kit (iNtRON biotechnology) with 0.02** **µg RNA per reaction containing 10 Pmol of specific primers, for 30 runs for 10** **sec of 95** **°C and 1** **min of 60** **°C. Threshold cycle or comparative Ct method was employed to establish the relative products amounts. The relative expression was computed employing the formula 2 (-ΔΔCt) (Mullou et al., 1995). They were scaled in relation to controls whereas control samples had a value of 1.

#### 2.5.7. Statistical analysis

Codes were set to the data and inputted employing the Graph Pad prism version 7 software. Differences between groups were verified by means of the Chi-Square test (qualitative variables), independent sample T-test, and analysis of variance (ANOVA). *Post hoc* Bonferroni test was used for normally distributed quantitative variables. The difference was considered a statistically significant when *P*-values were equal to or less than .05.

## 3. Results

### 3.1. Loratadine/sulpiride compatibility

#### 3.1.1. Fourier transform infrared spectroscopy

**Figure 1.**
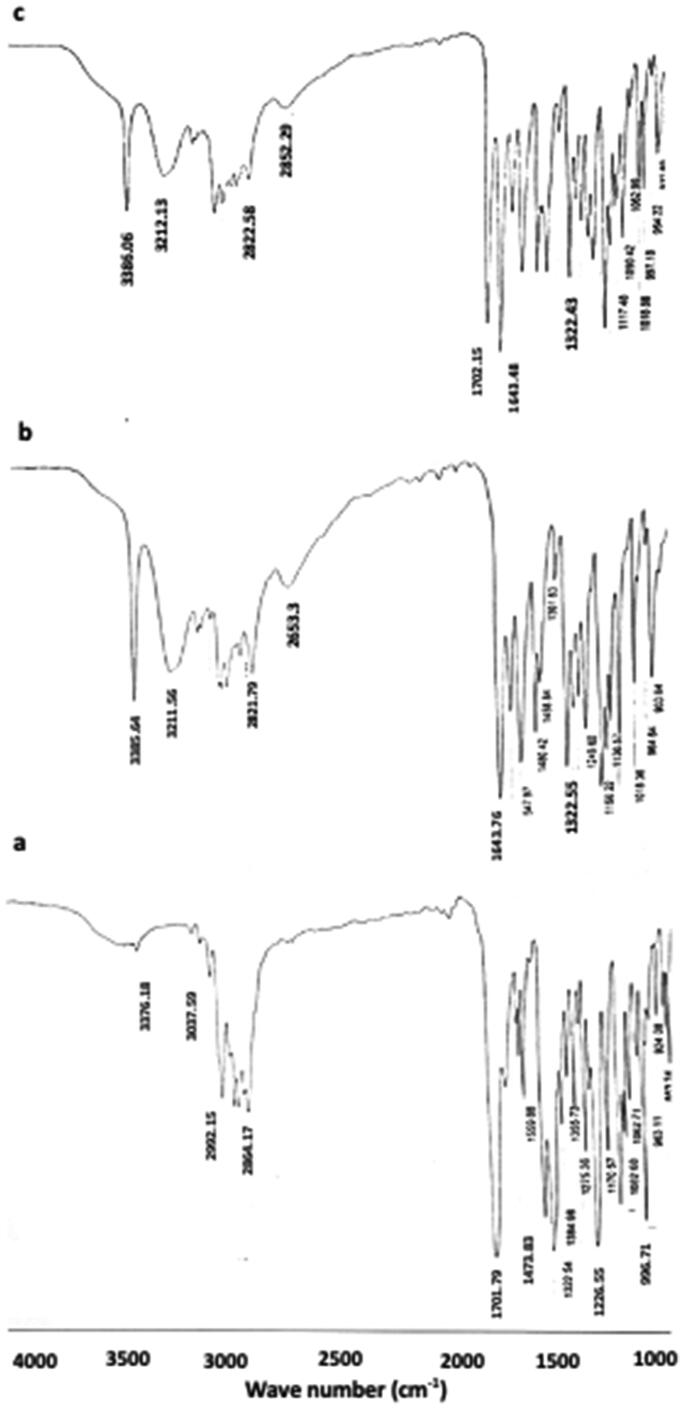
FTIR spectra of a. raw loratadine, b. raw sulpiride, c. 1:1 loratadine/sulpiride physical mixture.

The FTIR spectra of raw loratadine, sulpiride and their 1:1 physical mixture are displayed in Figure 1. Loratadine spectrum showed characteristic absorption bands in the range between 3,000 and 2,850** **cm^−1^ corresponding to C-H stretch. A strong peak appeared at 1,702** **cm^−1^ corresponding C = O group of ester. Other peaks were detected at 1,474 and 1,227** **cm^−1^ and 996** **cm^−1^ related to benzene ring stretching vibrations, C-H stretching and aryl C-Cl stretching respectively (Akhgari et al., 2016). The typical peaks of sulpiride were spotted at 3385** **cm^−1^ (N–H), 3211** **cm^−1^ (NH2), 1643** **cm^−1^ (C = O), and 1322** **cm^−1^ (SO2) (Zidan et al., 2015). The corresponding physical mixture spectrum displayed the presence of the typical peaks of both drugs.

#### 3.1.2. Differential scanning calorimetry study

**Figure 2.**
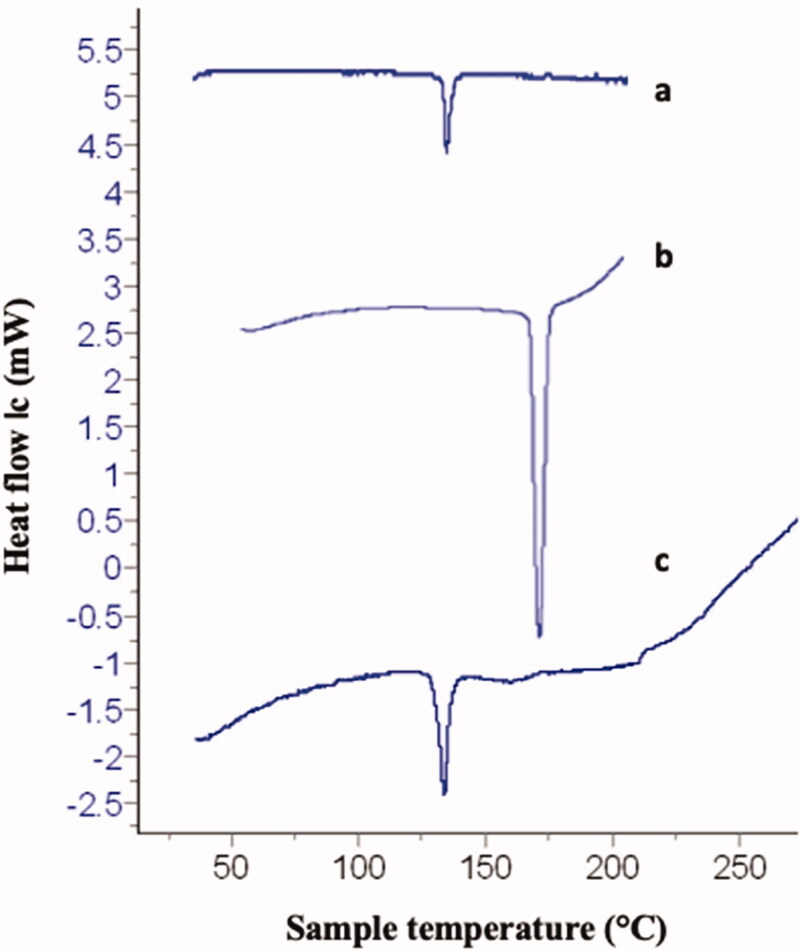
DSC thermograms of a. raw loratadine, b. raw sulpiride, c. 1:1 loratadine/sulpiride physical mixture.

DSC thermograms of raw loratadine, sulpiride and their 1:1 physical mixture are revealed Figure 2. Loratadine exhibited a sharp endotherm at 135 °C ascribed to its melting temperature Akhgari et al., 2016 (Akhgari et al., 2016). Thermogram of sulpiride displayed a sharp endotherm at a temperature of 175** **°C attributed to its melting transition (Zidan et al., 2015). The physical mixture DSC thermogram presented the melting endothermic peak of loratadine at 135** **°C while showed the absence of the melting endotherm of sulpiride.

### 3.2. Appearance, pH and viscosity

Appearance, pH and viscosity of different nanoemulsion formulations are displayed in Table 2. The resulting nanoemulsions were homogenous in appearance and did not display any indications for drug precipitation or phase separation. F1, F2 and F3 showed less cloudy appearance while F4, F5, F6 and F7 were cloudy. Viscosity of different nanoemulsion formulations ranged from 73.3** **±** **3.7 (F2) to 203** **±** **3.1 (F7). F6 and F7 had viscosity values that were significantly greater than those of the other formulations with no significant differences between their values (*p*** **>** **0.05). pH values were recorded in the range from 6.2** **±** **0.0 to 7.2** **±** **0.02.

**Table 2.** Appearance, pH, viscosity and %EE of the co-loaded loratadine/sulpiride nanoemulsion formulations.

Formulation code	Appearance	pH	Viscosity (mPa.s)	EE%	
				loratadine	sulipride
F1	Less cloudy	6.5 ± 0.01	80.8 ± 2.8	95.5 ± 0.1	95 ± 0.2
F2	Less cloudy	6.7 ± 0.02	73.3 ± 3.7	90 ± 0.2	93 ± 0.00
F3	Less cloudy	6.3 ± 0.00	81.2 ± 1.6	97.5 ± 0.00	98 ± 0.1
F4	Cloudy	7.2 ± 0.01	102.3 ± 2.1	96 ± 0.2	92 ± 0.02
F5	Cloudy	6.5 ± 0.1	108.5 ± 2.9	96 ± 0.02	93 ± 0.5
F6	Cloudy	6.2 ± 0.0	203 ± 3.1	93 ± 0.01	91 ± 0.2
F7	Cloudy	6.7 ± 0.02	201 ± 2.8	92 ± 0.3	92 ± 0.0

### 3.3. Entrapment efficiency

The EE% of the prepared nanoemulsions was investigated to determine the amount of loratadine and sulpiride loaded in the internal phase (oily phase) of these formulations and the results are displayed in Table 2. The EE% of the prepared nanoemulsions for both drugs exhibited high values more than 90% with insignificant differences between different formulations (*p*** **>** **.05). F3 displayed the highest values for loratadine (97.5** **±** **0.00) and sulpiride (98** **±** **0.1).

### 3.4. Droplet size, polydispersity index, zeta potential and electrical conductivity

Droplet size is an important characteristic for evaluation of the stability of nanoemulsion and improvement of drug bioavailability (Xi et al., 2009). It is an essential factor since it influences the drug release and biological absorption (Parul et al., 2013). Depending on the appearance, viscosity and entrapment results, F1, F2 and F3 were selected for this investigation. The mean droplet size, polydispersity index and zeta potential of these formulations are displayed in Table 3. F3 demonstrated the smallest droplet size (85.2** **±** **0.2** **nm) while F1 showed the largest one (149** **±** **2** **nm). F2 and F3 had the lowermost values of PDI (0.44** **±** **0.02 and 0.35** **±** **0.0 respectively) while F1 exhibited a PDI value of 0.78** **±** **0.01. Zeta Potential ranged from −20.8 to −29.7** **mV with F2 having the highest value. The electrical conductivity values ranged from 0.00 to 0.02 mS/cm. Thus, F2 and F3 were selected for following investigations.

**Table 3.** Droplet size, zeta potential, PDI and electrical conductivity of the selected nanoemulsions.

Formulation	Size (nm)	PDI	Zeta Potential (mV)	Conductivity (mS/cm)	% Intensity
F1	122.1 ± 1	0.78 ± 0.01	−24.1 ± 0.5	0.00 ± 0.00	94.6 ± 0.1
F2	102.7 ± 0.5	0.44 ± 0.02	−29.7 ± 0.2	0.01 ± 0.01	92.1 ± 0.2
F3	85.2 ± 0.2	0.35 ± 0.0	−23.3 ± 0.2	0.00 ± 0.00	84.7 ± 0.5

### 3.5. In vitro release studies

**Figure 3.**
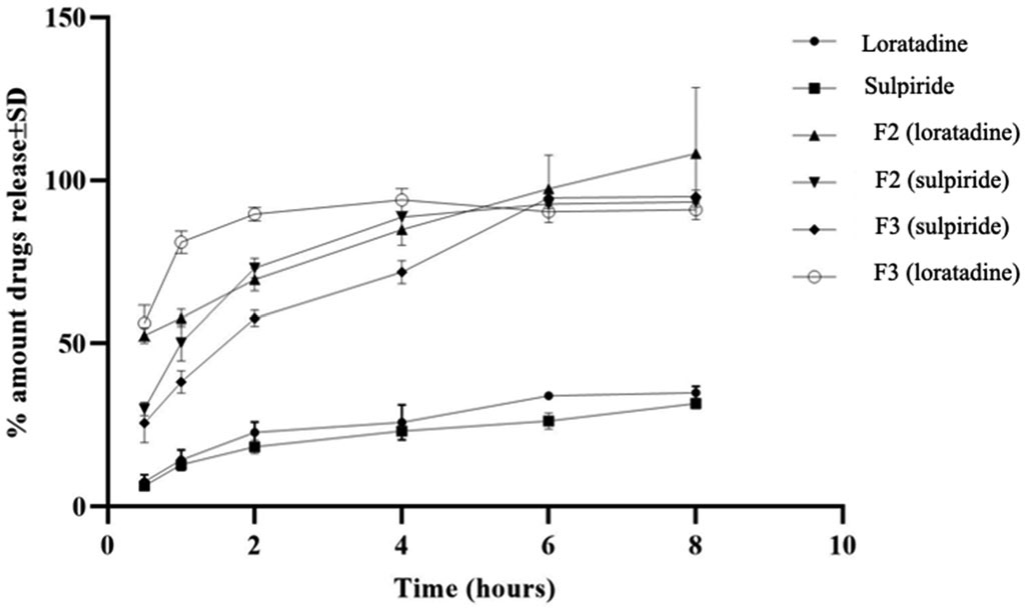
Release profiles of loratadine and sulpiride from the selected nanoemulsions, F2 and F3 compared to release profiles of the raw drugs (*n*** **=** **3).

This study was carried out to assess the release rate of loratadine and sulpiride from the selected nanoemulsions F2 and F3 in comparison to the raw drugs (Figure 3). The release of both drugs was enhanced from the investigated nanoemulsions displaying significantly higher values relative to those of the raw drugs (F_2_<50). The percentage amounts loratadine and sulpiride released after half an hour were 52.3%±.16 and 30%±0.3 from F2 or 58%±3 and 30%±2 from F3 respectively. On the other hand, the corresponding raw drugs showed 7%±1 and 6%±0.5 respectively at the same time interval. At the end of 8** **hours, the percentage amounts loratadine and sulpiride released were 98%±2 and 93%±0.1 from F2 or 95%±1.5 and 91%±2 from F3 while the raw drugs displayed 35%±0.1 and 30%±0.3 respectively. The difference between the release of loratadine and sulpiride from F2 and F3 was insignificant (F_2_>50). Table 4 shows the correlation coefficient (r) and release exponent (n) for the selected nanoemulsions (F2, F3). It is clear that the Higuchi’s diffusion and Baker–Lonsdale models had the highest correlation coefficient (r). Therefore, the release data of loratadine and sulpiride from the selected formulations was best fit to both models.

**Table 4.** Release kinetics of loratadine and sulpiride from the selected nanoemulsions.

Formulation	Loratadine					Sulpiride				
	Zero	First	Higuchi	Baker	Hixon	Zero	First	Higuchi	Baker	Hixon
F2	0.79	−0.79	0.98	0.99	0.79	0.79	−0.79	0.96	0.98	0.79
F3	0.79	−0.79	0.99	0.98	0.79	0.79	−0.79	0.92	0.85	0.79

### 3.6. Morphology analysis

**Figure 4.**
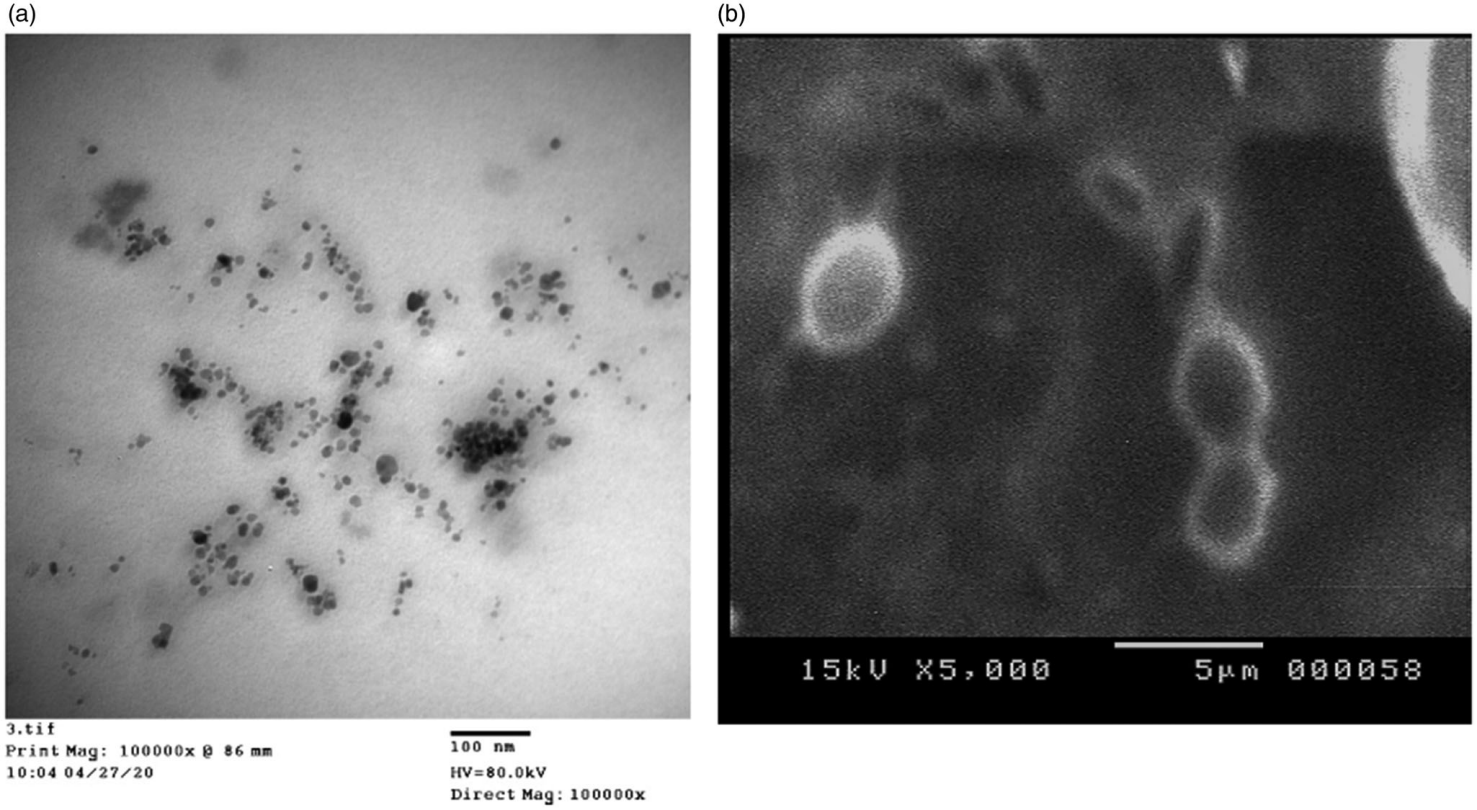
Transmission electron microscope (a) and scanning electron microscope (b) images of the selected nanoemulsion, F3.

The TEM and SEM images of the selected formulation, F3, were displayed in Figure 4 revealing spherical shape of the nanoemulsion droplets with the presence of some aggregations.

### 3.7. Stability studies

Stability studies for the optimized formulation F3 were conducted at 4** **°C and 25** **°C for one month, the results are illustrated in Table 5. Insignificant alterations were detected in the droplet size, PDI and entrapment efficiency of loratadine and sulpiride (*p*** **>** **.05). Additionally, the appearance of the studied nanoemulsion persisted with lower cloudiness and absence of phase separation till the end of the study time.

**Table 5.** Stability studies for F3 nanoemulsion at 4 °C and 25 °C.

Time (day)	4 °C				25 °C			
	Droplet size (nm)	PDI	EE (%)		Droplet size (nm)	PDI	EE (%)	
			Loratadine	Sulpiride			Loratadine	Sulpiride
0	135.2 ± 0.2	0.35 ± 0.0	97.5 ± 0.0	98 ± 0.1	135.2 ± 0.2	0.35 ± 0.0	97.5 ± 0.0	98 ± 0.1
15	138.2 ± 0.02	0.38 ± 0.1	97.3 ± 0.1	98 ± 0.1	141.2 ± 0.1	0.36 ± 0.0	98.1 ± 0.3	98 ± 0.1
30	137 ± 0.1	0.36 ± 0.0	96.6 ± 0.2	98 ± 0.1	144.2 ± 0.3	0.37 ± 0.2	97.1 ± 0.1	98 ± 0.1

### 3.8. *In vivo* efficacy study

#### 3.8.1. Allergic rhinitis symptoms

Table 6 displays the symptoms in different animal groups before and after the start of treatment with different formulations. Allergic rhinitis symptoms including nasal irritation and secretions, sneezing, eye secretions and conjunctivitis were found to gradually increase following induction procedures. The total score of the symptoms ranged from 11-12 before the start of treatment (day 1). After 14** **days of treatment with the co-loaded loratadine and sulpiride emulsion, these symptoms were significantly reduced (*p*** **<** **.05) displaying a total score of 6. Further reduction of the symptoms was detected after treatment with the co-loaded loratadine and sulpiride nanoemulsion with a total score of 3 (*p*** **<** **.001). On the other hand, Placebo group displayed non-significant reduction of the symptoms with a total score of 9 (*p*** **>.05).

**Table 6.** Evaluation of allergic rhinitis symptoms in rabbit models after treatment with different formulations for 14** **days.

	Negative control		Positive control		Nanoemulsion		Emulsion		Placebo	
	Day 1	Day 14	Day 1	Day 14	Day 1	Day 14	Day 1	Day 14	Day 1	Day 14
Nasal irritation	0	0	3	3	3	1	3	2	3	2
Sneezing	0	0	1	2	1	–	1	–	1	–
Nasal secretion	0	0	2	3	2	1	2	2	2	2
Eye secretions	0	0	3	3	3	1	2	1	3	3
Conjunctivitis	0	0	3	3	3	0	3	1	3	2
Total scores	0	0	12	14	12	3	11	6	12	9
*p*-Value					≤.001		≤.05		>.05	

#### 3.8.2. Relative mRNA expression

**Figure 5.**
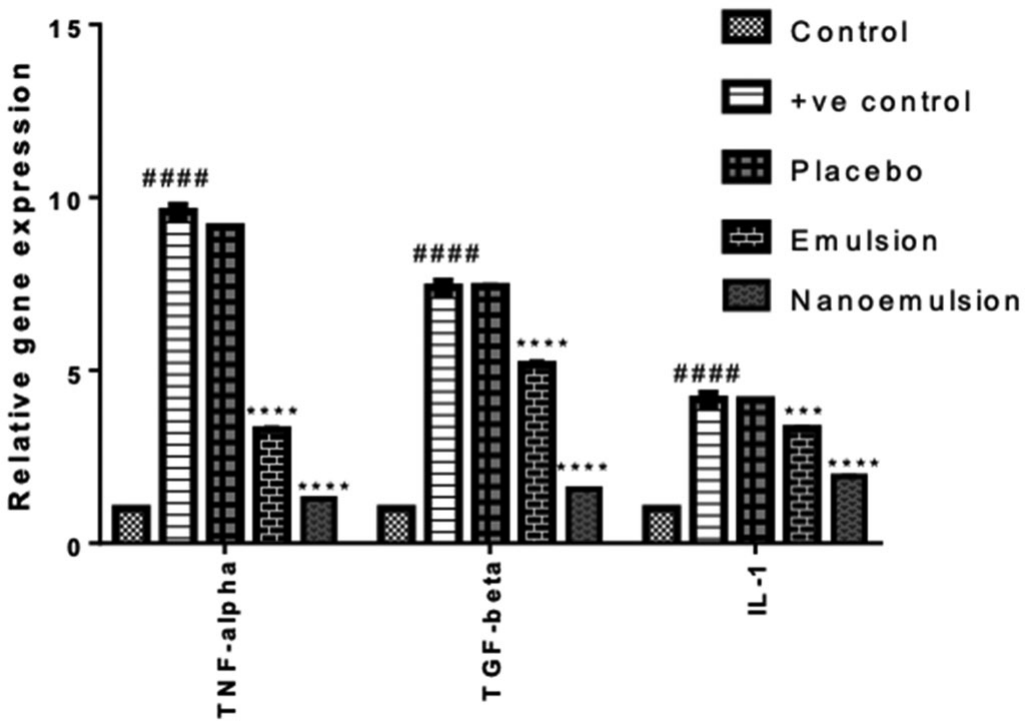
Relative expression of TNF-α, IL-1 and TGF-β in the study groups.

Table 7, Figure 5 show the mRNA expression of TNF-α, IL-1 and TGF-β after induction of allergic rhinitis and treatment with different formulations. The Nasal mucosa relative mRNA expression of TNF-α, IL-1 and TGF-β was significantly increased in positive control group (II) after induction of nasal mucosa inflammation relative to the negative control (I) (*p*** **<** **.0001). However, after treatment by placebo (III), the relative expression at the end of experiment showed no relative increase or decrease in the marker’s expression in comparison to the positive control (II). Instead, after treatment with the conventional emulsion co-loaded with loratadine and sulpiride (IV), the nasal mucosa relative mRNA expression of TNF-α, IL-1and TGF-β was significantly reduced relative to the positive control (II). However, nasal mucosa relative mRNA expression of TNF-α, IL-1and TGF-β in the group treated with the nanoemulsion formulation, F3 (V) displayed a further and highly significant decrease relative to the positive control (*p*** **<** **.0001).

**Table 7.** Mean relative gene expression of TNF-α, TGF-β and IL-1 in nasal mucosa of study groups.

	Negative control	Positive control	Placebo	Emulsion	Nanoemulsion
TNF-α	1.0	9.59 ± 1.06####	8.15 ± 0.07	3.28 ± 0.22****	1.28 ± 0.02****
TGF-β	1.0	7.4 ± 0.26####	7.41 ± 0.17	5.16 ± 0.31****	1.561 ± 0.02****
IL-1	1.0	4.15 ± 0.02####	4.13 ± 0.02	3.31 ± 0.18***	1.934 ± 0.06****

*p*** **>** **.05: non-significant (ns), *p*** **<** **.05: mild significant (*), *p*** **<** **.01: significant (**), *p*** **<** **.001: highly significant (***), *p*** **<** **.0001: very highly significant (****) and *p*** **<.0001: very highly significant (####) by T-test unpaired.

## 4. Discussion

FTIR and DSC have been employed to investigate the possibility of incidence of interaction between loratadine and sulpiride. The existence of the characteristic FTIR absorption bands of both loratadine and sulpiride in the spectrum of the corresponding physical mixture (at the same positions compared to the spectra of the individual drugs) refers to the absence of drug/drug interaction. The presence of the melting endotherms of loratadine in the physical mixture DSC thermogram revealed the nonexistence of drug interaction supporting the FTIR results. The absence of melting endotherm of sulpiride could be attributed to the dissolution of the drug in the melt of loratadine as the two drugs have close melting temperatures. The less cloudy appearance of F1, F2 and F3 compared to other formulations could be related to diminished droplet size resulting in reasonably weak scattering rendering the nanoemulsion system optically translucent (McClements, 2002; McClements, 2002). The higher viscosity values of F6 and F7 relative to other formulations might be ascribed to the higher concentration of lecithin or glycerol respectively. Similarly, Zhou et al., have revealed a rise in the viscosity of a lecithin nanoemulsion by the increase in the concentration of lecithin or glycerol (Zhou et al., 2009). The high entrapment efficiency of loratadine and sulpiride in the prepared nanoemulsions could be attributed to increased drug solubility in the crude olive which contains a combination of unsaturated fatty acids that provide a cosolvent effect (Balata et al., 2016). This result could also be ascribed to the high ester value of olive oil (190.86) indicating high percentage of ester groups (Zambiazi et al., 2007). This value is an indication for the proportion of glycerol existing in the oil that gives high solubilizing capacity for the oil (Azeem et al., 2009). In a recent study, olive oil has been reported to have the uppermost solubilizing capacity for the hydrophobic drug gliclazide relative to other screened oils (Balata, 2018). These results are also in accordance with a preceding work that revealed that olive oil has a good solubilizing ability for the hydrophobic drug resveratrol (Balata et al., 2016). In addition, the hydrophilic nonionic surfactant, Tween 80, having HLB 15 might maximize the solubilizing power, which is essential for affording a uniform emulsion. Furthermore, lecithin as a natural emulsifying agent might enhance the solubilization of both drugs in the oil and hence entrapment efficiency. The resulted small droplet size is important for drug bioavailability as it leads to greater surface area for drug absorption. The small droplet size of F3 compared to F1 and F2 could be ascribed to the higher total concentration of the used surfactants (Tween 80 and sodium cholate) that might jacket the surfaces of the new droplet produced throughout homogenization and lower the oil/water interfacial tensions (Samson et al., 2016). It has been reported that the average droplet size of nanoemulsion reduces with the increase in concentration of surfactant due to the formation of larger water-oil interface (Joung et al., 2016). Furthermore, combination of hydrophilic surfactant Tween 80 and lecithin (natural emulsifying agent) decreases the interfacial tension during emulsification process and consequently reduces the nanoemulsion droplet size (Guttoff et al., 2015). The small PDI values of F2 and F3 indicates narrow globule size distribution, which reflects uniformity in the size distribution and droplet diameter of both formulations (Balakumar et al., 2013). The measured zeta potentials reflect good stability of the selected systems with F2 having the highest stability. Generally, it has been reported that zeta potential of ± 30** **mV was appropriate for nanoemulsion stability; (Müller et al., 2001; Balakumar et al., 2013). Formulations with high zeta potential have higher stability, as they resist coalescence of oil droplets through enhancing the electrostatic repulsion between the charged globules (Balakumar et al., 2013). The electrical conductivity of the selected nanoemulsions revealed good quality nanoemulsions and support stability (Sari et al., 2015). The enhanced release of loratadine and sulpiride from F2 and F3 compared to the corresponding raw rugs could be ascribed to the small droplet size of the investigated nanoemulsions which imparts large surface area for release (Alshehri et al., 2020). Thus, both drugs existed in solubilized micellar solution which greatly enhanced their release from the selected nanoemulsions (Balata, 2018). Kinetic of release of loratadine and sulpiride from F2 and F3 obeyed the Higuashi Diffusion model denoting that there was a direct proportional relationship between the amount of drug release and either the square root of the total amount of the drugs or the drug solubility in the nanoemulsion formulation (Sarpal et al., 2010). Also, the release kinetics followed the model of Baker–Lonsdale that was established from Higuchi model and described the release of drugs from the sphere-shaped matrices. Analogous findings have been documented for the release of chlorehexidine HCL from a promising antibacterial root canal irrigant nanoemulsion (Abdelmonem et al., 2019). Stability study revealed that F3 maintained excellent physical stability at 4 °C and 25 °C for one month.

The high significant reduction of the allergic rhinitis symptoms that was observed after treatment with the co-loaded loratadine and sulpiride nanoemulsion revealed a promising effect that was superior to that of the corresponding emulsion formulation. In response to actuation by extraneous particles, macrophages discharge TNF-α, a motivator of the inherent immune response. In a paracrine way, TNF-α instigates adjoining cells to generate interleukin-8 (to provoke phagocyte staffing) (Yao et al., 2005) and E-selectin (to support adhesion of phagocytes to the adjacent endothelium) (Hermosilla et al., 2006). Autocrine activity by TN-Fα encourages further TNF-α production and initiate macrophages to produce and release IL-1. In a paracrine way, IL-1 provokes the localized formation of Interleukin-6 and the expression of Intercellular Adhesion Molecule-1 (ICAM-1). The overall products of protein produced by TNFα and IL-1 acting together to facilitate the inherent immune response and induce the adaptive immune response (Cottam et al., 2004; Ott et al., 2007). TGF-β has a responsibility in numerous processes involving regeneration of epithelial cells, inflammation and healing of tissue. TGF-β plays an essential role in the initial immune response as it acts as a chemoattractant and activator of inflammatory cells. It also fosters downregulation of inflammation across impediment of actuated cells and stimulation of apoptosis exerting anti-inflammatory effects (Otto & Wenzel, 2008).

The significant increase of the nasal mucosa relative mRNA expression of TNF-α, IL-1 and TGF-β in positive control group after induction of nasal mucosa inflammation indicated that the induction of inflammation was successful resulting in upregulation of this expression. The absence of change in the marker’s expression after treatment by free drug nanoemulsion indicated that the treatment with the plain nanoemulsion had no effect on these markers. The significant decrease of the marker’s expression after treatment with co-loaded loratadine and sulpiride conventional emulsion indicated that the treatment with the emulsion causes downregulation of these marker’s expression. However, the further and highly significant decrease of these markers after treatment with the nanoemulsion formulation, F3, revealed a superior downregulation of the inflammatory markers. Thus, the co-loaded loratadine and sulpiride nanoemulsion had a more efficacious action in managing inflammation compared to the corresponding emulsion formulation.

## 5. Conclusion

In the present work, co-loaded loratadine and sulpiride nanoemulsions were developed using soybean lecithin and olive oil in addition to surfactants. F3 that displayed small droplet size, low PDI, applicable Zeta Potential and enhanced in vitro drug release was selected as the prime nanoemulsion. The loratadine and sulpiride release of from F3 followed the Higuashi Diffusion and Baker–Lonsdale models. F3 presented a good stability over a month upon storage at 4** **°C and 25** **°C. The biological study revealed enhanced downregulation of inflammatory parameters; TNF-α, TGF-β and IL-1 in rabbit models of ovalbumin-induced allergic rhinitis compared to corresponding emulsion formulation. These findings support that the co-loaded loratadine and sulpiride nasal nanoemulsion as a new approach can provide an encouraging effect in handling the symptoms of allergic rhinitis.
